# Pilot Investigation of SARS-CoV-2 Secondary Transmission in Kindergarten Through Grade 12 Schools Implementing Mitigation Strategies — St. Louis County and City of Springfield, Missouri, December 2020

**DOI:** 10.15585/mmwr.mm7012e4

**Published:** 2021-03-26

**Authors:** Patrick Dawson, Mary Claire Worrell, Sara Malone, Sarah C. Tinker, Stephanie Fritz, Brett Maricque, Sadaf Junaidi, Gemille Purnell, Albert M. Lai, Julie A. Neidich, Justin S. Lee, Rachel C. Orscheln, Rachel Charney, Terri Rebmann, Jon Mooney, Nancy Yoon, Machelle Petit, Spring Schmidt, Jean Grabeel, Lee Ann Neill, Lisa C. Barrios, Snigdha Vallabhaneni, Randall W. Williams, Clay Goddard, Jason G. Newland, John C. Neatherlin, Johanna S. Salzer, CDC COVID-19 Surge Laboratory Group, CDC COVID-19 Surge Laboratory Group, Suxiang Tong, Ying Tao, Brian Emery, Jing Zhang, Min-hsin Chen, Gimin Kim, Bettina Bankamp

**Affiliations:** ^1^CDC COVID-19 Response Team; ^2^Epidemic Intelligence Service, CDC; ^3^Washington University in St. Louis, St. Louis, Missouri; ^4^Saint Louis University, St. Louis, Missouri; ^5^Springfield–Greene County Health Department, Springfield, Missouri; ^6^Saint Louis County Department of Public Health, Berkeley, Missouri; ^7^Springfield Public Schools, Springfield, Missouri; ^8^Missouri Department of Health and Senior Services.

Many kindergarten through grade 12 (K–12) schools offering in-person learning have adopted strategies to limit the spread of SARS-CoV-2, the virus that causes COVID-19 (*1*). These measures include mandating use of face masks, physical distancing in classrooms, increasing ventilation with outdoor air, identification of close contacts,* and following CDC isolation and quarantine guidance^†^ (*2*). A 2-week pilot investigation was conducted to investigate occurrences of SARS-CoV-2 secondary transmission in K–12 schools in the city of Springfield, Missouri, and in St. Louis County, Missouri, during December 7–18, 2020. Schools in both locations implemented COVID-19 mitigation strategies; however, Springfield implemented a modified quarantine policy permitting student close contacts aged ≤18 years who had school-associated contact with a person with COVID-19 and met masking requirements during their exposure to continue in-person learning.^§^ Participating students, teachers, and staff members with COVID-19 (37) from 22 schools and their school-based close contacts (contacts) (156) were interviewed, and contacts were offered SARS-CoV-2 testing. Among 102 school-based contacts who received testing, two (2%) had positive test results indicating probable school-based SARS-CoV-2 secondary transmission. Both contacts were in Springfield and did not meet criteria to participate in the modified quarantine. In Springfield, 42 student contacts were permitted to continue in-person learning under the modified quarantine; among the 30 who were interviewed, 21 were tested, and none received a positive test result. Despite high community transmission, SARS-CoV-2 transmission in schools implementing COVID-19 mitigation strategies was lower than that in the community. Until additional data are available, K–12 schools should continue implementing CDC-recommended mitigation measures (*2*) and follow CDC isolation and quarantine guidance to minimize secondary transmission in schools offering in-person learning.

A student, teacher, or staff member who received a positive SARS-CoV-2 nucleic acid amplification test or antigen test result and who had been physically present at the school or a school-associated event while potentially infectious was most often reported to school officials within 1–2 days of receipt of laboratory results. School officials initiated contact tracing to identify contacts in the school environment^¶^ within 12–24 hours of notification. In Springfield, school officials assessed whether student contacts met criteria for a modified quarantine based on information from the contact tracing investigation. During December 7–18, 2020, an investigation team from Washington University in St. Louis, Saint Louis University, and CDC invited eligible** persons with COVID-19 and their contacts to participate in the pilot investigation within 12–24 hours of identification by school officials. Overall numbers of identified contacts in the school environment were available for analysis regardless of participation and were used to characterize the number of school contacts identified per case.

To collect more detailed contact tracing information and epidemiologic data, a trained interviewer conducted a standardized telephone interview with 1) participants aged ≥18 years, 2) participants aged 12–17 years and/or their parents or guardians, and 3) parents or guardians of participants aged <12 years. Data were entered into and managed in a REDCap database (version 9.5.5; Washington University in St. Louis) and analyzed using SAS (version 9.4; SAS Institute). Contacts were monitored prospectively until 14 days after their last exposure. Saliva samples were collected from persons with COVID-19 soon after they agreed to participate and from contacts 5–8 days after their last exposure; samples were tested for SARS-CoV-2 by reverse transcription–polymerase chain reaction (RT-PCR).^††^ Whole genome sequencing (97%–99% coverage) was conducted on RT-PCR–positive saliva samples using Oxford Nanopore Technologies MinION sequencing at CDC (*3*). For each contact who received a positive SARS-CoV-2 RT-PCR test result, the investigation team followed a case determination protocol to ascertain whether school-based secondary transmission was probable, possible, or unlikely^§§^ (*4*,*5*). To gather data on mitigation measures implemented in schools, standardized interviews were conducted with school officials representing 57 K–12 schools (12 St. Louis County schools and 45 Springfield schools). This project was reviewed and approved by the Washington University in St. Louis Institutional Review Board and by CDC and was conducted consistent with applicable federal law and CDC policy.^¶¶^

All schools offered in-person learning, and all but one offered full- or part-time virtual learning. Among all schools, 9,216 of 30,558 (30%) students were participating in virtual learning only, and 21,342 (70%) attended in-person school at least part-time. Data on implemented mitigation strategies were reported for 55 schools, and 100% implemented a mask mandate. In addition, in at least some classrooms, 100% of schools spaced desks ≥3 ft apart, 27% spaced desks ≥6 ft apart, and 98% placed physical barriers between teachers and students. Ninety-eight percent had handwashing or hand sanitizing stations available at school entrances, and 100% had stations available in cafeterias or other dining areas, restrooms, and classrooms. Modifications to increase ventilation to prevent COVID-19 were reported by 98% of schools: 91% opened windows or doors, 87% used fans, 93% decreased occupancy in spaces where ventilation with outdoor air could not be increased, and 5% replaced or updated heating, ventilation, and air conditioning systems.

School officials identified 56 persons with COVID-19 who had a total of 270 contacts with school-based exposure and monitored them until the end of their isolation or quarantine period (Figure). All 326 persons were eligible for participation in the pilot investigation (interview, saliva testing, or both); among these, 193 (59%) agreed to participate. Participants included 37 (66%) persons with COVID-19 and 156 (58%) contacts from 22 of the 57 participating schools. Among participating persons with COVID-19 and their contacts, 65% and 88%, respectively, were students. Distributions by gender, age, and race/ethnicity among participating persons with COVID-19 and contacts were similar (Table). The number of identified contacts per participating person with COVID-19 ranged from 1 to 35 (median = 5).

**FIGURE:**
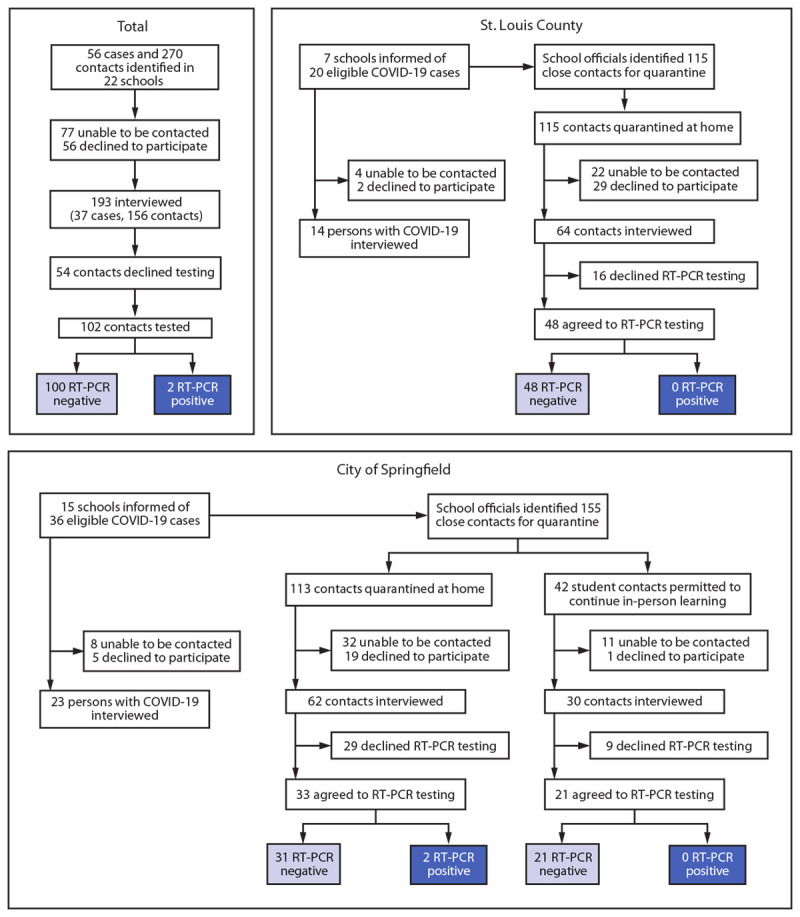
Identification of students, teachers, and staff members with school-associated COVID-19,* school-based close contacts,^†^ and SARS-CoV-2 RT-PCR test results^§^ among close contacts — St. Louis County and city of Springfield, Missouri,^¶^^,^** December 2020 **Abbreviations:** K–12 = kindergarten through grade 12; NAAT = nucleic acid amplification test; RT-PCR = reverse transcription–polymerase chain reaction. * Receipt of a positive NAAT or antigen test result in a student, teacher, or staff member who was physically present at the school or a school-associated event while potentially infectious; cases were most often reported to school officials within 1–2 days of laboratory results. ^†^ Any person who spent a cumulative total of ≥15 minutes in one 24-hour period within 6 ft of a person with COVID-19 while that person was potentially infectious, regardless of mask use. A person with COVID-19 was considered potentially infectious to others starting from 2 days before symptom onset (or if asymptomatic, 2 days before the collection of their first positive SARS-CoV-2 test specimen) until the person was isolated. https://www.cdc.gov/coronavirus/2019-ncov/php/contact-tracing/contact-tracing-plan/appendix.html#contact ^§^ Among 168 contacts who did not receive testing from the investigation team, during the 14 days after their last exposure, no other school-associated cases with a positive SARS-CoV-2 NAAT or antigen test result were reported to school officials. ^¶^ In November 2020, Springfield–Greene County Health Department and Springfield Public Schools adopted a modified quarantine policy for K–12 schools. Under this policy, student close contacts of a person with COVID-19 were permitted to attend school in person during their quarantine period if 1) the school had a mask mandate, the school’s classrooms were arranged to maximize physical distancing, the school had increased hand hygiene practices, and the school screened students and staff members for COVID-19 symptoms and immediately isolated symptomatic persons and 2) the close contacts were K–12 students aged ≤18 years, their only exposure to the person with COVID-19 was in the educational environment (e.g., a classroom), they did not have prolonged (≥15 minutes) direct physical contact with the person with COVID-19, and the close contacts and person with COVID-19 had all been wearing masks appropriately during the time of exposure. https://www.springfieldmo.gov/5369/Modified-Quarantine ** The two close contacts who received positive SARS-CoV-2 RT-PCR test results were from separate Springfield schools, were quarantining at home, and were contacts of two different persons with COVID-19 (persons A and B). School-based secondary transmission was probable for both contacts based on their exposure histories and symptom and testing timelines. One student contact of person A (a student in the same grade) received a positive test result 6 days after exposure. Although no genetic sequencing data were available, the student had no other known sources of exposure. One student contact of person B (a teacher) received a positive SARS-CoV-2 test result 7 days after exposure. The student was exposed in the classroom (<3 ft from the teacher for >15 minutes) and had no other known exposure sources. The consensus sequence generated from whole genome sequencing of the student’s saliva sample was nearly identical to that of person B, differing by only one nucleotide.

**TABLE:** Characteristics of persons with school-associated COVID-19 cases* and their school-based close contacts^†^ from 22 kindergarten through grade 12 schools — St. Louis County and city of Springfield, Missouri, December 2020

Characteristic	No. (%)		
	Cases (n = 37)	Contacts (n = 156)	Total (n = 193)
**School location**			
St. Louis County^§^	14 (38)	64 (41)	78 (40)
City of Springfield^¶^	23 (62)	92 (59)	115 (60)
**Age of students, yrs, median (range)**	14 (6–18)	11 (5–18)	12 (5–18)
**Age of teachers/staff members, yrs, median (range)**	50 (29–61)	44 (28–63)	47 (28–63)
**School status**			
Elementary school student (grades K–5)	7 (19)	65 (42)	72 (37)
Middle school student (grades 6–8)	4 (11)	21 (13)	25 (13)
High school student (grades 9–12)	13 (35)	51 (33)	64 (33)
Teacher	7 (19)	12 (8)	19 (10)
Staff member	6 (16)	7 (4)	13 (7)
Gender identity**			
Female	22 (59)	88 (56)	110 (57)
Male	15 (41)	65 (42)	80 (41)
Other/Nonbinary	0 (—)	2 (1)	2 (1)
Unknown	0 (—)	1 (1)	1 (1)
**Race**			
American Indian or Alaska Native	1 (3)	1 (1)	2 (1)
Asian	0 (—)	1 (1)	1 (1)
Black	3 (8)	29 (19)	32 (17)
Native Hawaiian or Other Pacific Islander	0 (—)	0 (—)	0 (—)
White	26 (70)	115 (74)	141 (73)
Multiracial	4 (11)	6 (4)	10 (5)
Prefer not to say or unknown	3 (8)	4 (3)	7 (4)
**Ethnicity**			
Hispanic/Latino	3 (8)	11 (7)	14 (7)
Non-Hispanic/Latino	34 (92)	142 (91)	176 (91)
Prefer not to say or unknown	0 (—)	3 (2)	3 (2)
**Preexisting medical condition^††^**			
Yes	17 (46)	49 (31)	66 (34)
No	20 (54)	105 (67)	125 (65)
Unknown	0 (—)	2 (1)	2 (1)

* Receipt of a positive nucleic acid amplification test or antigen test result in a student, teacher, or staff member who was physically present at the school or a school-associated event while potentially infectious; cases were most often reported to school officials within 1–2 days of laboratory results.

^†^ Any person who spent a cumulative total of ≥15 minutes in one 24-hour period within 6 ft of a person with COVID-19 while that person was potentially infectious, regardless of mask use. A person with COVID-19 was considered potentially infectious to others starting from 2 days before symptom onset (or if asymptomatic, 2 days before the collection of the first positive SARS-CoV-2 test specimen) until the person was isolated. https://www.cdc.gov/coronavirus/2019-ncov/php/contact-tracing/contact-tracing-plan/appendix.html#contact

^§^ Includes participants from seven schools.

^¶^ Includes participants from 15 schools.

** In response to the question “I am going to read a list of genders. Please let me know which one you identify yourself as (your child identifies themselves as).”

^††^ Including but not limited to type 1 or type 2 diabetes, hypertension, cardiovascular disease, chronic renal disease, chronic lung disease, immunosuppressive conditions, autoimmune conditions, and premature birth.

Fifty-four of the 156 participating contacts declined testing; among the 102 who were tested, two (2%) received positive SARS-CoV-2 test results (Figure). These two contacts were from separate schools in Springfield and were contacts of two different persons with COVID-19 (persons A and B) (5% of participating persons with COVID-19). School-based secondary transmission was probable for both contacts based on their exposure histories and symptom and testing timelines. One student contact of person A (a student in the same grade) received a positive test result 6 days after exposure. Although no sequencing data were available, the student contact had no other known sources of exposure. One student contact of person B (an elementary school teacher) received a positive SARS-CoV-2 test result 7 days after exposure in the classroom (<3 ft for >15 minutes) and had no other known exposure sources. The consensus sequence generated from whole genome sequencing of the student’s saliva sample was nearly identical to that of person B, differing by only one nucleotide. Because neither contact of person A or B who received a positive test result met the criteria for Springfield’s modified quarantine, they completed their quarantine at home.*** Of the 168 contacts who did not receive testing from the investigation team, none was identified by school officials as having received positive test results during the 14 days after their last school-based exposure.

In the Springfield school district that implemented a modified quarantine, 131 (85%) of 155 contacts were students, 82 (63%) of whom agreed to participate in the pilot investigation; 42 (51%) participants met criteria for a modified quarantine and continued in-person learning during their quarantine period, 30 (71%) of whom were interviewed. Among 52 student contacts who did not meet modified quarantine criteria and were interviewed, the most common reasons student contacts did not meet modified quarantine criteria were unmasked exposure (31; 60%), athletic activity contact (11; 21%), and lunch or recess contact (seven; 13%). Testing results were available for 21 (70%) of 30 students who participated in the modified quarantine and were interviewed, and none received a positive SARS-CoV-2 test result.

## Discussion

Schools across the United States have adopted various strategies to limit the risk for SARS-CoV-2 transmission and reduce disruptions to in-person learning (*1*). In-person school has psychosocial and health benefits beyond educational enrichment for many children, particularly those who depend on school-based services for physical, nutritional, and mental health support (*6*). Various mitigation strategies were implemented by the 55 surveyed schools with available data, including face mask mandates, increased physical distancing in classrooms, use of physical barriers to separate teachers from students, increased ventilation with outdoor air, and virtual learning options.

In this 2-week pilot investigation in K–12 schools that had implemented multiple strategies to limit SARS-CoV-2 transmission, school-based secondary transmission involving 37 participating students, teachers, and staff members with COVID-19 was identified among only two (2%) of 102 tested school close contacts. In both instances of probable school-based secondary transmission, each person with COVID-19 infected only one other person in the school environment. No outbreaks were identified in participating schools, despite the 2-week cumulative community incidence of 711 COVID-19 cases per 100,000 persons in St. Louis County^†††^ and 996 in Springfield–Greene County.^§§§^ Considering that only two probable school-based secondary transmission cases were identified, the 2-week school incidence would have been approximately eight cases per 100,000 persons, <1% of the average community incidence in the two sites over the same time period.^¶¶¶^ These findings are consistent with other studies that have reported that despite high community SARS-CoV-2 transmission, schools that implemented multicomponent measures to reduce spread reported lower in-school transmission (*7*,*8*) unless lapses in these measures occurred (*9*).

The Springfield school district, which implemented a modified quarantine for certain students, permitted 42 student contacts to continue in-person learning during their quarantine period; 30 of these contacts were interviewed, and none of the 21 students who received testing had a positive test result. Assuming that an average 10-day quarantine period**** results in 8 missed school days, an estimated 240 person-days of in-person learning were saved by implementing the modified quarantine for these student contacts. However, the testing data for participating student contacts in modified quarantine are insufficient to recommend that other schools nationwide adopt a modified quarantine policy; additional data are needed.

The findings in this report are subject to at least five limitations. First, school contact tracing might not have identified all close contacts. Second, participation in the investigation project was 59%, possibly because of COVID-19 testing fatigue or other factors cited by persons who declined to participate. Third, not all possible mitigation strategies or school characteristics that might have affected school-based secondary transmission were assessed, and whether masks were worn appropriately was not assessed. Fourth, symptom status, mitigation practices, exposure histories, and underlying medical conditions of persons who participated might have differed from those who did not. Therefore, these findings might not be representative of all persons in the participating schools and might not be generalizable to other schools. Finally, persons who were infected with SARS-CoV-2 but who did not receive testing in this investigation might have been missed, particularly if they were asymptomatic and did not receive testing elsewhere.

The findings from this pilot investigation suggest that implementation of CDC-recommended SARS-CoV-2 mitigation measures in schools might help reduce school-based transmission. The absence of positive test results among student contacts who participated in a modified quarantine raises important epidemiologic questions that require additional study, including the effect of modified quarantine on school-based SARS-CoV-2 secondary transmission and specific criteria for a modified quarantine. The pilot investigation did not include sufficient data to answer these questions; however, data from a more extensive ongoing investigation in six urban, suburban, and rural Missouri public school districts representing approximately 70,000 students, teachers, and staff members will help address these important public health concerns. Until additional data are available, K–12 schools should continue implementing SARS-CoV-2 mitigation strategies that include mask use policies, physical distancing, increased ventilation, and attention to hand hygiene (*2*) and follow CDC isolation and quarantine guidance to minimize secondary transmission of SARS-CoV-2 in schools offering in-person learning.

**Summary**

**What is already known about this topic?**

Many kindergarten through grade 12 (K–12) schools have implemented strategies to limit school-associated SARS-CoV-2 transmission.

**What is added by this report?**

In 22 participating K–12 schools implementing multiple COVID-19 mitigation strategies, school-based SARS-CoV-2 secondary transmission was detected in two of 102 tested close contacts of 37 persons with COVID-19. Among 21 tested student contacts participating in a modified quarantine, all SARS-CoV-2 test results were negative.

**What are the implications for public health practice?**

Schools implementing strategies including mask mandates, physical distancing, and increased ventilation had much lower SARS-CoV-2 transmission than in the community. K–12 schools should continue implementing these measures and following CDC isolation and quarantine guidance to minimize secondary transmission in schools.

## Footnotes

* A close contact was defined as any person who spent a cumulative total of ≥15 minutes in one 24-hour period within 6 ft of a person with COVID-19 while that person was potentially infectious, regardless of mask use. A person with COVID-19 was considered potentially infectious to others starting from 2 days before symptom onset (or if asymptomatic, 2 days before the collection of the first positive SARS-CoV-2 test specimen) until the person was isolated. https://www.cdc.gov/coronavirus/2019-ncov/php/contact-tracing/contact-tracing-plan/appendix.html#contact (accessed February 4, 2021).

† https://www.cdc.gov/coronavirus/2019-ncov/if-you-are-sick/quarantine.html (accessed January 31, 2021).

§ In November 2020, Springfield–Greene County Health Department and Springfield Public Schools adopted a modified quarantine policy for K–12 schools. Under this policy, student close contacts of a person with COVID-19 were permitted to attend school in person during their quarantine period if 1) the school had a mask mandate, the school’s classrooms were arranged to maximize physical distancing, the school had increased hand hygiene practices, and the school screened students and staff members for COVID-19 symptoms and immediately isolated symptomatic persons and 2) the close contacts were K–12 students aged ≤18 years, their only exposure to the person with COVID-19 was in the educational environment (e.g., a classroom), they did not have prolonged (≥15 minutes) direct physical contact with the person with COVID-19, and the close contacts and person with COVID-19 had all been wearing masks appropriately during the time of exposure. https://www.springfieldmo.gov/5369/Modified-Quarantine (accessed January 2, 2021).

¶ The school environment includes exposures inside or outside of the classroom and school-based extracurricular and athletic activities.

** Persons with COVID-19 who were present in the school environment while potentially infectious ≤5 days before recruitment and contacts whose most recent exposure was ≤5 days before recruitment were eligible for inclusion. Participants aged ≥18 years provided oral agreement to participate; parent/guardian oral agreement was required for children aged <18 years, and oral agreement was required from children aged 12–17 years. Contacts could participate in the investigation regardless of whether the person with COVID-19 to whom they were exposed participated; however, contacts were excluded if they lived with the person who had COVID-19.

†† Saliva samples were tested at Washington University in St. Louis using the Washington University SARS-CoV-2 Ultrasensitive-High-Throughput-Saliva Version 1.0 assay. This RT-PCR assay is designed to detect two regions of the N gene (N1 and N2) in SARS-CoV-2 using primers and probes that were developed and validated under the Emergency Use Authorization for the CDC assay, as well as endogenous human control RNA (RNase P). Rarely, a health care provider collected a sample via nasal swab instead of a saliva sample from a contact to test for SARS-CoV-2 by RT-PCR outside of the pilot investigation, and results were reported to the investigation team.

§§ Case determinations were made as follows: 1) probable school-based transmission: the person who received a positive SARS-CoV-2 test result was a close contact of someone with laboratory-confirmed COVID-19 in the school environment only, had no other known exposure to another person with confirmed or suspected COVID-19 in the 14 days before symptom onset or date of collection of the first specimen with positive test results, the person’s exposure history and symptom and testing timeline was consistent with the known epidemiology of COVID-19 (e.g., did not experience symptoms on the same day as the first contact with the person with COVID-19), and (if sequencing data were available) the sequences generated from the specimens from the contact and school index case were identical or nearly identical; 2) possible school-based transmission: the person who received a positive SARS-CoV-2 test result was a close contact of a person with laboratory-confirmed COVID-19 in the school environment but had nonhousehold community exposure to that person or another person with confirmed or suspected COVID-19 in the 14 days before symptom onset or date of collection of their first specimen with positive test results, the person’s exposure history and symptom and testing timeline was consistent with the known epidemiology of COVID-19, and sequencing data were unavailable or indeterminate; 3) unlikely school-based transmission: the person who received a positive SARS-CoV-2 test result was a close contact of a person with laboratory-confirmed COVID-19 in the school environment but lived in the same household as another person with confirmed or suspected COVID-19 in the 14 days before symptom onset or date of collection of the first specimen with positive test results or the person’s exposure history and symptom and testing timeline was not consistent with the known epidemiology of COVID-19 and (if sequencing data were available) the sequences generated from the contact’s specimen and the school index case’s specimen were not identical or nearly identical.

¶¶ 45 C.F.R. part 46, 21 C.F.R. part 56; 42 U.S.C. Sect. 241(d); 5 U.S.C. Sect. 552a; 44 U.S.C. Sect. 3501 et seq.

*** Neither contact met the criteria for modified quarantine: one had contact at lunch and recess in addition to in the classroom with person A and the other had prolonged direct physical contact with person B during one-on-one instruction. Because both close contacts did not meet the criteria for modified quarantine, both were completing their respective quarantine periods at home.

††† Data from https://stlcorona.com/resources/covid-19-statistics/archived-covid-19-reports/archived-trend-reports/covid-19-trends-12-28-2020/ (accessed January 1, 2021).

§§§ Data from https://www.springfieldmo.gov/5147/Recovery-Dashboard (accessed January 1, 2021).

¶¶¶ Crude incidence calculation based an estimated 26,000 in-person students, teachers, and staff members in participating schools over a 2-week period, compared with 711 (St. Louis County) and 996 (Springfield–Greene County) cases per 100,000 persons (combined average of 854 cases per 100,000 persons).

**** https://www.cdc.gov/coronavirus/2019-ncov/more/scientific-brief-options-to-reduce-quarantine.html (accessed February 12, 2021).
